# Two-stage maximum likelihood approach for item-level missing data in regression

**DOI:** 10.3758/s13428-020-01355-x

**Published:** 2020-04-24

**Authors:** Lihan Chen, Victoria Savalei, Mijke Rhemtulla

**Affiliations:** 1 University of British Columbia, Vancouver, Canada; 2 University of California Davis, Davis, CA USA

**Keywords:** Missing data, Item level, Regression, Two stage

## Abstract

Psychologists use scales comprised of multiple items to measure underlying constructs. Missing data on such scales often occur at the item level, whereas the model of interest to the researcher is at the composite (scale score) level. Existing analytic approaches cannot easily accommodate item-level missing data when models involve composites. A very common practice in psychology is to average all available items to produce scale scores. This approach, referred to as available-case maximum likelihood (ACML), may produce biased parameter estimates. Another approach researchers use to deal with item-level missing data is scale-level full information maximum likelihood (SL-FIML), which treats the whole scale as missing if any item is missing. SL-FIML is inefficient and it may also exhibit bias. Multiple imputation (MI) produces the correct results using a simulation-based approach. We study a new analytic alternative for item-level missingness, called two-stage maximum likelihood (TSML; Savalei & Rhemtulla, *Journal of Educational and Behavioral Statistics, 42*(4), 405–431. 2017). The original work showed the method outperforming ACML and SL-FIML in structural equation models with parcels. The current simulation study examined the performance of ACML, SL-FIML, MI, and TSML in the context of univariate regression. We demonstrated performance issues encountered by ACML and SL-FIML when estimating regression coefficients, under both MCAR and MAR conditions. Aside from convergence issues with small sample sizes and high missingness, TSML performed similarly to MI in all conditions, showing negligible bias, high efficiency, and good coverage. This fast analytic approach is therefore recommended whenever it achieves convergence. R code and a *Shiny* app to perform TSML are provided.

## Introduction

Psychologists across many subfields often use measures that are composed of multiple items. For example, the UCLA Loneliness Scale (Russell, 1996) has 20 items, and the Big Five Inventory has 8–10 items on each personality dimension. The composite scale scores computed from these items are frequently used in analyses such as regression. This application is found in a wide range of research topics from the relationship between personality types and depression (Dhondt et al., 2013), mind-wandering and attention deficit disorder (Seli, Smallwood, Cheyne, & Smilek, 2015), to internet and smartphone addiction (Choi et al., 2015).

When participants filling out a scale answer some but not all items, the result is item-level missing data. Participants answering an inventory questionnaire may refuse to answer questions that they deem too sensitive, leave items blank when they do not apply, quit the questionnaire early because it is too long, or skip items due to carelessness. Item-level missing data presents a particularly difficult problem when the researcher is interested in fitting a model at the composite level, which requires thecomputation of composite scores, because it is not straightforward to compute such scores in the presence of missing data.

It is common practice for psychologists to deal with item-level missing data by simply taking the means of available items. This approach is equivalent to person-mean imputation and is also known as *proration* (Mazza, Enders, & Ruehlman, 2015) or available-case analysis (Savalei & Rhemtulla, 2017). An alternative is to treat the composite score as missing entirely, with all the available items deleted, which we refer to as scale-level deletion. In reality, researchers often use a hybrid approach of computingscale scores based on all available items when their number is above a threshold, and declaring the scale score as missing otherwise. The threshold for this hybrid strategy can vary greatly from one researcher to another. For example, Culbert, Breedlove, Sisk, and Burt (2013) computed scale scores based on all available cases for scales missing 10% of the items or less, and treated the whole scale as missing when more than 10% of the items were missing, whereas Beebe et al. (2007) applied the same procedure at 50% of the items missing. While methodologists have long known that such procedures are theoretically unsound (Schafer & Graham, 2002), simulation studies that investigate the extent of the bias and under what conditions it arises have been scarce. In this study, we compare our proposed approach to both available-case analysis and scale-level deletion.

Several recent studies have compared the performance of different item-level missing data techniques (Orcan, 2013; Parent, 2013; Mazza et al., 2015; Savalei & Rhemtulla, 2017). However, until recently, *item-level multiple imputation* (item-level MI; Rubin, 1987) remained the only statistically justified approach that can deal with item-level missing data. Multiple imputation fills in (*imputes*) the missing values based on the existing data, but incorporating a random component into each imputed value, often based on distributional assumptions. This procedure is repeated multiple times to arrive at *m* complete datasets. The statistical analysis of interest is then performed on each imputed dataset, and the estimates are aggregated across imputations. Because it is a simulation-based approach, MI has properties that are not desired by some researchers. It can be cumbersome and difficult to implement for applied researchers, and it can take a long time, depending on the specific MI method and the number of imputations requested. Further, when MI is performed on the same dataset multiple times, it may produce a slightly different answer every time. The results may also be sensitive to parameters in the implementation of the method such as the number of imputations performed.

Outside of the context of item-level missing data, MI under the normal model is largely equivalent to an analytical approach known as *full-information maximum likelihood* (FIML). However, FIML cannot easily handle item-level missing data when the model is at the composite level (but see Rose, Wagner, Mayer, & Nagengast, 2019, for a new approach). In this article, we propose and study a more flexible analytical alternative, called two-stage maximum likelihood (TSML; Yuan & Bentler, 2000; Yuan & Lu, 2008; Savalei & Bentler, 2009). TSML has recently been extended to handle item-level missing data (Savalei & Rhemtulla, 2017). The original work on TSML showed good performance of the method with item-level missing data that occurs in the context of structural equation models (SEMs) with parcels. However, SEMs with latent variables are quite different from statistical models many researchers ordinarily use. For instance, they require large sample sizes, and may perform quite differently in small samples. SEMs also assume a reflective rather than a formative model of measurement, requiring that the set of items conforms to a certain structure (Rhemtulla, van Bork, & Borsboom, in press). At the same time, researchers commonly use composites made up of scale items or other behavioral indicators with small samples and in simpler analyses such as regression. The current study adapts the TSML approach to the context of basic univariate regression, and examines its performance under a large array of simulation conditions, including small sample sizes that are more typical in regression settings. For simplicity, we focus on normally distributed data, but the extension of the TSML appraoch to nonnormal data is straightforward and will be considered in future studies.

### Missing data mechanisms

Rubin (1987) defined three types of missing data mechanisms: missing completely at random (MCAR), missing at random (MAR), and missing not at random (MNAR). Under MCAR, the probability of missingness on any variable is independent of any variable in the dataset, whether missing or observed. Under MAR, the probability of missingness cannot depend on variables with missing values, conditioning on variables that have been observed. Under MNAR, missingness depends on variables with missing data even after conditioning on complete variables. With an additional mild assumption of the independence between model parameters and the parameters guarding missing data, MCAR and MAR are known as *ignorable* missing data mechanisms (Little & Rubin, 2002), because modern missing data techniques such as FIML and MI can deal with these types of missing data well, resulting in accurate and efficient estimates. MNAR, on the other hand, is always nonignorable, so it is much more difficult to handle. Because dealing with MNAR requires explicit modeling of the missing data mechanism on a case by case basis (for example, see Galimard, Chevret, Protopopescu, & Resche-Rigon, 2016), studies of general techniques for dealing with missing data are typically limited to the study of the ignorable mechanisms, MCAR and MAR.

### Item-level missing data techniques

#### Available-case maximum likelihood

Available-case maximum likelihood (ACML) is a popular technique for dealing with item-level missing data (Mazza et al., 2015) in an SEM context. When a scale has missing item scores, the ACML method simply takes the mean of all available items for each participant as their scale score; the usual maximum likelihood (ML) on the resulting complete data composites is then performed to fit the SEM.^1^ Obtaining the scale mean from all available cases is the same as performing person-mean imputation; that is, each participant’s missing values within each scale are replaced by the mean of that participant’s scores on the available items on that scale.

Disadvantages of person-mean imputation are well known. When handling item-level missing data, ACML produces incorrect standard errors by assuming there is no missing data. ACML also tends to produce biased mean estimates and to underestimate relationshipbetween scales under MAR missingness. ACML may produce biased results even under MCAR (Schafer & Graham, 2002; Mazza et al., 2015). However, ACML may produce reasonable parameter estimates in some situations. For example, Mazza et al. (2015) have shown that if the intercorrelations of all the items are the same within each scale, and the item means are the same within the scale, the ACML parameter estimates may be unbiased under MAR. Even in such an ideal scenario, the ACML standard errors may be inaccurate. More importantly, it is often the case that the assumptions of equal item means and equal item correlations are untenable. Despite its disadvantages, ACML is undoubtedly convenient. As a result, researchers may be tempted to assume equal intercorrelations and equal item means in order to use it. Even under these ideal conditions, methodologists recommend this approach only for small amounts of missing data (<10%) (Parent, 2013). The current study will examine the performance of ACML in the context of univariate regression in a wide range of conditions.

#### Scale-level full-information maximum likelihood

Full-information maximum likelihood (FIML) is a modern method for analyzing missing data that produces consistent parameter estimates under an MAR mechanism. When data are missing at the item level, scale-level FIML (SL-FIML; Savalei & Rhemtulla, 2017) is the approach that uses listwise deletion to compute scale scores followed by FIML at the composite level. That is, for each participant, if any item is missing, the whole associated scale is treated as missing. This approach would clearly result in a significant power loss. However, there is a far more insidious problem: if the items that the MAR mechanism depends on (i.e., items that predict missingness) are deleted as part of the initial listwise deletion, the missing mechanism becomes MNAR. Under MNAR, SL-FIML is likely to produce biased estimates.

It has been shown that SL-FIML is sensitive to properties of the scale. For example, SL-FIML may produce relatively unbiased estimates when all items within each scale have the same means across all participants, but biased estimates when item means differ (Mazza et al., 2015). Mazza et al. (2015) aimed to address the bias by using some of the items as auxiliary variables in the model. However, all items cannot be used as auxiliary variables in the composite model due to the resulting linear dependencies among the variables in the model, e.g., if a scale has 5 items, only 4 can be used as auxiliary variables. Recently, Rose et al. (2019) have proposed an ingenious way to implement item-level FIML for a composite-level model in SEM; however, this approach may be cumbersome to implement and requires further study.

#### Multiple imputation

Multiple Imputation (MI; Rubin, 1976; Little, 2017) is another advanced modern method for handling MAR missingness. Unlike analytical methods such as FIML, MI produces estimates using a numerical, simulation-based approach. MI involves three stages: imputation, analysis, and pooling. During the imputation stage, MI duplicates the data, and performs random single imputations on each duplicated dataset independently. The imputation stage produces multiple complete datasets that differ from each other due tothe randomness in the single imputations. MI then performs the intended data analysis on each complete dataset. Finally, MI pools parameter estimates and standard errors across the datasets using Rubin’s rules (Rubin, 1987). It is straightforward to handle item-level missingness using MI, because the composites can easily be computed within each complete dataset.

Although MI produces consistent and highly efficient estimates, the ideal number of imputations necessary to achieve good results may be surprisingly large in some situations (Graham, Olchowski, & Gilreath, 2007). Performing a large number of imputations requires a long computation time, which can render the approach less accessible to some researchers. The approach may also be difficult to understand and implement for applied researchers, as there may be advanced settings necessary to specify for running the imputations. Finally, because it is a simulation-based approach, the estimates and standard errors produced by MI will be slightly different if the procedure is repeated, or if a different version of the procedure is implemented. In general, fast analytical approaches that produce a unique set of estimates are preferred to MI, when they are possible.

#### Two-stage maximum likelihood

In this article, we study an alternative analytical approach, known as two-stage maximum likelihood (TSML). The original TSML approach is a more flexible alternative to FIML that is applicable to any situation where there is missing data (Savalei & Bentler, 2009). TSML involves estimating the saturated means and covariances model (using FIML) for all the variables in Stage 1, and then fitting the desired model (e.g., regression or SEM) to the summary statistics obtained from Stage 1 (i.e., means and covariances) as if they were obtained from complete data. Doing so requires the computation of robust standard errors for the model parameter estimates, because the “naive” standard errors that would be produced by the software in Stage 2 assume the data are complete. When the data are multivariate normal, TSML is not as efficient as FIML, but the loss of efficiency is small. The TSML approach has been modified to handle incomplete nonnormal data (Yuan & Bentler, 2000; Yuan & Lu, 2008) and to use auxiliary variables (Savalei & Falk, 2014), where it has been shown to do at least as well as, and sometimes outperform, FIML. TSML with normal and nonnormal data has been implemented in the R package *lavaan* (Rosseel, 2012) under the options *estimator = “ML”*, *method = “two.stage”*.

Recently, Savalei and Rhemtulla (2017) introduced an extension of the TSML method to handle item-level missing data. Its chief advantage over ACML and scale-level FIML is that it does not require first performing listwise deletion or person-level imputation at the item level. Further, TSML for item-level data would be preferred to item-level MI because it is a fast analytical approach that produces unique estimates. For a large number of imputations, TSML and item-level MI are expected to be equivalent. Savalei and Rhemtulla (2017) studied the performance of TSML in the context of SEMs with parcels. Here, we adapt the TSML extension to the case where the composite-level model is a simple regression model. While we study simple univariate regression, the method is easily generalizable to any number of predictors, or to more complicated models such as path analysis. Because the TSML extension to item-level missing data was developed in the SEM context, in order to apply the TSML approach to regression models, they will be fit as saturated models via SEM software. Because this approach is new, it has not yet been implemented in SEM software, and we provide R code a Shiny app for its use. Technical details for the TSML extension to item-level missing data arenow given.

Let $X={\left({X}_1,{X}_2,\dots, {X}_{p_1}\right)}^{\prime }$ be the items on the predictor composite, and let $Y={\left({Y}_1,{Y}_2,\dots, {Y}_{p_2}\right)}^{\prime }$ be the items on the outcome composite. All variables are then represented as a *p* × 1 vector, *Z* = (*X*^′^, *Y*^′^)^′^, where *p* = *p*_1_ + *p*_2_. Let *X*_*c*_ and *Y*_*c*_ be the sum scores for the two scales, respectively, and *Z*_*c*_ = (*X*_*c*_, *Y*_*c*_)^′^. When the model contains *m* composite variables, $p={\sum}_{i=1}^m{p}_i$, and *Z*_*c*_ is an *m* × 1 vector. While the current study only involves the case of *m* = 2, the generalized description is included for completeness. During Stage 1, the saturated model is fit to the *p* items contained in *Z* to obtain FIML estimates of the population parameters, which for the saturated model are simply the *p* × 1 vector of means, ${\hat{\mu}}_p$, and the *p* × *p* covariance matrix, ${\hat{\Sigma}}_p$. Let ${\hat{\gamma}}^{\prime }=\left(\mathrm{vech}{\hat{\Sigma}}_p,{\hat{\mu}}^{\prime}\right)$ be the (*p*^∗^ + *p*) × 1 vector of the saturated model parameter estimates, where ${p}^{\ast }=\frac{1}{2}p\left(p+1\right)$ and the “vech” operator selects the nonredundant elements of a covariance matrix columnwise (Magnus & Neudecker, 1989). From this saturated FIML solution, we also obtain the associated *p*^∗^ × *p*^∗^ observed information matrix, ${\hat{A}}_{\gamma }$, and its inverse, ${\hat{\Omega}}_{\gamma }={\hat{A}}_{\gamma}^{-1}$, which is an estimate of the asymptotic covariance matrix of $\hat{\gamma}$ under multivariate normality.

An additional step, Stage 1a, is now necessary to convert the item-level components from Stage 1 to scale-level components. To perform the conversion, we define an *m* × *p* selection matrix *C* such that *Z*_*c*_ = *CZ*. For example, for two composites containing 3 items each, $$ C=\left(\begin{array}{cccccc}1& 1& 1& 0& 0& 0\\ {}0& 0& 0& 1& 1& 1\end{array}\right) $$. The corresponding saturated model estimates of the means and the covariance matrix of *Z*_*c*_ are given by ${\hat{\mu}}_c=C\hat{\mu}$, an *m* × 1 vector, and ${\hat{\Sigma}}_c=C{\hat{\Sigma}}_p{C}^{\prime }$, a *m* × *m* matrix. We stack these saturated estimates into a single vector, $$ \hat{\delta}=\left(\begin{array}{c}\mathrm{vech}{\hat{\Sigma}}_c\\ {}{\hat{\mu}}_c\end{array}\right) $$. We then define an additional transformation matrix *C*_S_, obtained from *C* as follows: $$ {C}_{\mathrm{S}}\left(\begin{array}{cc}{D}_m^{+}\left(C\bigotimes C\right){D}_p& 0\\ {}0& C\end{array}\right) $$,where D_*p*_ is the duplication matrix of order *p*, and ${D}_m^{+}$ is the Moore-Penrose inverse of the duplication matrix of order *m* (Magnus & Neudecker, 1989). Then, scale-level saturated estimates are related to the item-level saturated estimates via the equation, $\hat{\delta}={C}_{\mathrm{S}}\hat{\gamma}$. Finally, the associated asymptotic covariance matrix of $\hat{\delta}$ is obtained from the item-level asymptotic covariance matrix via the equation, ${\hat{\Omega}}_{\delta }={C}_S{\hat{\Omega}}_{\gamma }{C}_S^{\prime }$.

During Stage 2, the scale-level model is fit to the estimated means and covariance matrix of *Z*_*c*_ from Stage 1a, i.e., ${\hat{\mu}}_c$ and ${\hat{\Sigma}}_c$, as if the underlying data were complete. Let *θ* represent the *q* × 1 vector of SEM model parameters, which are hypothesized to structure the population means and covariance matrix of *Z*_*c*_, i.e., Σ_*c*_(*θ*) and *μ*_*c*_(*θ*). To obtain parameter estimates, we minimize the complete data ML fit function:

$$ {F}_{\mathrm{ML}}\left(\theta \right)=\mathrm{tr}\left\{{\hat{\Sigma}}_c{\Sigma}_c^{-1}\left(\theta \right)\right\}-\log \mid {\hat{\Sigma}}_c{\Sigma}_c^{-1}\left(\theta \right)\mid +{\left({\hat{\mu}}_c-{\mu}_c\left(\theta \right)\right)}^{\prime }{\Sigma}_c^{-1}\left(\theta \right)\left({\hat{\mu}}_c-{\mu}_c\left(\theta \right)\right)-m. $$

Let the resulting TSML estimates be $\overset{\sim }{\theta }$, and the corresponding estimates of means and covariances under the hypothesized model ${\overset{\sim }{\mu}}_C={\mu}_C\left(\overset{\sim }{\theta}\right) and{\overset{\sim }{\Sigma}}_C={\Sigma}_C\left(\overset{\sim }{\theta}\right)$. A consistent estimate of the asymptotic covariance matrix of $\overset{\sim }{\theta }$, accounting for missing data, is given by the “sandwich” estimator,

$$ {\overset{\sim }{\Omega}}_{\theta }={\left({\overset{\sim }{\Delta}}^{\prime}\overset{\sim }{H}\overset{\sim }{\Delta}\right)}^{-1}{\overset{\sim }{\Delta}}^{\prime}\overset{\sim }{H}{\hat{\Omega}}_{\delta}\overset{\sim }{H}\overset{\sim }{\Delta}{\left({\overset{\sim }{\Delta}}^{\prime}\overset{\sim }{H}\overset{\sim }{\Delta}\right)}^{-1}, $$

where $\overset{\sim }{\Delta}={\left.\frac{\partial \delta \left(\theta \right)}{\partial {\theta}^{\prime }}\right|}_{\theta =\overset{\sim }{\theta }}$ is the matrix of model derivatives evaluated at $\overset{\sim }{\theta }$, and

$$ \overset{\sim }{H}=\left(\begin{array}{cc}.5{D}_m^{\prime}\left({\overset{\sim }{\Sigma}}_c^{-1}\otimes {\overset{\sim }{\Sigma}}_c^{-1}\right){D}_m& 0\\ {}0& {\overset{\sim }{\Sigma}}_c^{-1}\end{array}\right) $$

is the normal theory weight matrix evaluated at $\overset{\sim }{\theta }$, which is also the “naive” information matrix from Stage 2. The matrix product ${\left({\overset{\sim }{\Delta}}^{\prime}\overset{\sim }{H}\overset{\sim }{\Delta}\right)}^{-1}$ is the “naive” covariance matrix of parameter estimates that would be produced by default under complete-data ML estimation (Yuan & Bentler, 2000). The expression for the asymptotic covariance matrix of $\overset{\sim }{\theta }$ given above works for any general SEM. However, when the hypothesized model is also saturated, as is the case with any regression model viewed as an SEM, $\overset{\sim }{\Delta}$ is a square invertible matrix, and the expression can be simplified to:

$$ {\overset{\sim }{\Omega}}_{\theta }={\left(\overset{\sim }{\Delta}\right)}^{-1}{\overset{\sim }{H}}^{-1}{\left({\overset{\sim }{\Delta}}^{\prime}\right)}^{-1}{\overset{\sim }{\Delta}}^{\prime}\overset{\sim }{H}{\hat{\Omega}}_{\delta \prime}\overset{\sim }{H}\overset{\sim }{\Delta}{\overset{\sim }{\Delta}}^{-1}{\overset{\sim }{H}}^{-1}{\left(\overset{\sim }{\Delta}\prime \right)}^{-1}={\left(\overset{\sim }{\Delta}\right)}^{-1}{\hat{\Omega}}_{\delta }{\left(\overset{\sim }{\Delta}\prime \right)}^{-1} $$

In other words, when applying TSML to regression, all that is needed to compute accurate estimates of standard errors are the scale-level asymptotic covariance matrix from Stage 1a and the matrix of model derivatives, which captures the one-to-one transformation between the default saturated model of SEM (i.e., unrestricted variances and covariances for all observed variables) and the saturated model parameterized as a regression model.

### Previous simulation studies

The development of the TSML approach can be traced back to Yuan and Bentler (2000). Motivated by the need to deal with nonnormal missing data in SEM, these authors developed asymptotically correct standard errors and test statistics for FIML and TSML under MCAR nonnormal data. The MCAR assumption was required because consistency of FIML estimates under nonnormality had not yet been established. Yuan and Bentler (2000) compared FIML and TSML in a simulation study with sample sizes of *N* = 1000 and *N* = 2000, on two variables with varying degree of normality. MCAR missingness was generated by removing data on half the cases, with a 50% sample missing rate. MAR missingness was generated by removing all values corresponding to the top 50% of the conditioning variable, with a 50% population missing rate. Across all distributional conditions, FIML and TSML performed similarly under MCAR and MAR, and further did not show bias under MAR (Yuan & Bentler, 2000).

Yuan (2009) showed mathematically that FIML retains the property of consistency under MAR with nonnormal data, as long as the variables are linearly related to each other. Yuan and Lu (2008) and Savalei and Bentler (2009) extended and studied the the TSML approach with MAR data. Savalei and Bentler (2009) thoroughly evaluated TSML and FIML in a simulation study with normal data, and Savalei and Falk (2014) considered nonnormal data. TSML and FIML performed similarly across a range of sample sizes, percent missing data, and types of missing data mechanisms, with TSML showing a slight advantage with nonnormal data.

Few studies have investigated methods for item-level missing data via simulation. Mazza et al. (2015) investigated the performance of several approaches for item-level missingness in the context of univariate regression, where each composite had either 8 or 16 items, with sample sizes of 200 and 500. MAR was created using logistic regression with *R*^2^ = .4, and for 5%, 15%, and 25% per variable missing rate. A reflective model was assumed for the items. In the equal loading conditions, all loadings were .75 (implying item inter-correlations of .56). In the unequal loading conditions, loadings for items without missing data were set to .5 instead (implying item inter-correlations of .25 among some items). In the equal means conditions, item means were all 0. In the unequal mean conditions, the means of the items were set to .5 when missing values were present, and set to 0 otherwise. The study found that when either the item inter-correlations or item means varied within the same scale, ACML resulted in biased estimates of the regression coefficients. This study also included an approximation to the FIML approach where as many items as possible were added to the composite model to serve as auxiliary variables. This approach outperformed ACML.

Finally, Savalei and Rhemtulla (2017) proposed and studied the TSML approach to item-level missing data in the context of SEMs with parcels. The study compared ACML, SL-FIML, MI, and TSML on data generated from a second-order CFA model with 3 s-orderand 9 first-order factors. The composite model was a 3-factor model with 9 variables, which were parcels formed from the 27 indicators of the first-order factors. Fourteen out of the 27 original variables contained missing data with 5%, 15%, or 30% missing per variable. The study found that factor loadings were biased under some types of MAR for both ACML and SL-FIML, but were not biased for TSML or MI, as would be theoretically expected.

The goals of the current simulation study are threefold: 1) to empirically confirm that TSML performs well in the context of univariate regression, and in particular that its performance matches that of item-level MI; 2) to investigate the performance of TSML with ordinal as well as continuous data, and 3) to illustrate the disadvantages of ACML and SL-FIML yet again, as these methods remain popular among applied researchers. To facilitate the use of TSML by applied researchers, we also provide R code anda *Shiny* app with its implementation.^2^

## Method

### Design overview

The current study investigated ACML, SL-FIML, MI, and TSML in the context of univariate regression. We constructed 8 population models, defined by differences in the value of the regression coefficient, item intercorrelations, and intercepts. Both the predictor, *X* and the dependent variable, *Y*, were composites comprised of 8 items. The missing mechanisms studied were MCAR, strong linear MAR, weak linear MAR, strong nonlinear MAR, and weak nonlinear MAR. These mechanisms will be described below. Either 15% or 25% of the values on half of the items in each composite were missing, resulting in an overall missingness rate of 7.5% and 12.5%. Sample sizes of 50, 100, and 200 were studied. We also included three statistical variable types: continuous, binary, and ordinal with 4 levels. In total, ACML, SL-FIML, and TSML analyses were conducted on 5 mechanisms × 3 sample sizes × 2 missing rates × 8 population models × 3 variable types = 720 simulation conditions. Additionally, MI simulations were conducted on all 240 continuous variable conditions, as well as a subset of the binary variable conditions (Model 1 & 8, which contain 60 conditions in total).

### Continuous item conditions

The regression model fit to data was *Y*_*c*_ = *α* + *βX*_*c*_ + *e*, where composite scores *X*_*c*_ and *Y*_*c*_ were means of the item sets *X*_1_, …, *X*_8_ and *Y*_1_, . …, *Y*_8_, respectively. It is worth noting that while ACML and SL-FIML explicitly compute these composite scores, TSML does not. Instead, TSML uses the individual items directly to obtain Stage 1 means and covariances, and then converts those to scale-level means and covariances to be used in Stage 2 (via Stage 1a), thus fitting the model to scale-level information without ever needing to compute *X*_*c*_ and *Y*_*c*_.

All items were drawn from a multivariate normal distribution with all item variances equal to 1. Items *X*_1_, …, *X*_4_ each had a mean of *μ*_1_, with all item intercorrelations of *ρ*_1_. Items *X*_5_, …, *X*_8_ each had a mean of *μ*_2_, with all itemintercorrelations of *ρ*_2_. The correlation between each of *X*_1_, …, *X*_4_ and each of *X*_5_, …, *X*_8_ was set to $\sqrt{\rho_1{\rho}_2}$. Items in *Y*_*c*_ had the same structure as items in *X*_*c*_. Finally, let ${\sigma}_{X_c}$ be the standard deviation of the composite score *X*_*c*_, then the correlation (or covariance) between any pair of individual *X*_*i*_ and *Y*_*j*_ was is related to the unstandardized^3^ regression coefficient *β* and the standarddeviation of the composite scale as follows: ${\rho}_{xy}=\beta {\sigma}_{X_c}^2$.

In the equal item mean conditions, *μ*_1_ = *μ*_2_ = 0. In the unequal mean conditions, *μ*_1_ = 0, and *μ*_2_ = .5. In the equal item intercorrelation conditions, *ρ*_1_ = *ρ*_2_ = .49. This corresponded to the scale reliability of .88 (for both *X* and *Y* items). In unequal intercorrelation conditions, *ρ*_1_ = .25 and *ρ*_2_ = .64. This corresponded to the scale reliability of .86. However, in the unequal inter-correlation conditions, the correlation structure of both sets of items was notconsistent with a two-factor model, so measurement may be best thought of as formative.

In the equal intercorrelation conditions, *ρ*_*xy*_ = .37 was used to produce conditions with high regression coefficients, and *ρ*_*xy*_ = .22 was used to produce conditions with medium regression coefficients. In order to maintain roughly equivalent *ρ*_*xy*_s and *β*s between equal vs unequal intercorrelation conditions, *ρ*_*xy*_ = .32 and *ρ*_*xy*_ = .18 were used to produce the high and medium regression correlation conditions, respectively. Thus, we arrived at a list of 8 models (2 × 2 × 2) by manipulating the regression coefficient (medium, high), intercept (equal, unequal), and item intercorrelations (equal, unequal). The exact model parameters are summarized Table 1.

**Table 1:** Parameters of each population model

Model	*ρ*_1_	*ρ*_2_	*μ*_1_	*μ*_2_	*β*	*α*
1	.49	.49	0	0	.40	0
2	.49	.49	0	.5	.40	.15
3	.25	.64	0	0	.37	0
4	.25	.64	0	.5	.37	.16
5	.49	.49	0	0	.67	0
6	.49	.49	0	.5	.67	.08
7	.25	.64	0	0	.65	0
8	.25	.64	0	.5	.65	.09

*Note. ρ*_1_ and *ρ*_2_ denote the scale intercorrelations. *μ*_1_, *μ*_2_ denote the item means. *β* is the slope of the scale level regression, and *α* is the intercept of the scale level regression

### Discrete item conditions

In empirical research, items are typically ordinal. While a Likert item with 5–7 categories can be reasonably approximated by a continuous variable (Rhemtulla, Brosseau-Liard, & Savalei, 2012), the performance of the missing data techniques developed for continuous data may suffer when the ordinal items contain fewer categories. In order to compare TSML and other methods under these conditions, we created the binary conditions and 4-level ordinal conditions by discretizing continuous items, which were generated using the same methods described above. For binary item conditions, items were dichotomized at each item’s population mean, *μ*. Values that were lower than the item’s population mean were assigned 1, and 2 otherwise. For ordinal items with 4 levels, two additional thresholds were added at ±1 standard deviation away from the mean (i.e., *μ* − 1 and *μ* + 1). Note that as long as the predictor and the outcome variable use the same scale when converted to numeric values, the population value of the unstandardized regression coefficient *β* is unaffected by which scale we choose. Missingness was subsequently assigned based on the continuous data prior to discretization, using methods described below.

### Missing data mechanisms

For all missing data mechanisms, item *X*_5_ was used as the conditioning variable for all *X*_*i*_ variables that were missing. Similarly, *Y*_5_ was used as the conditioning variable for missing *Y*_*i*_ variables. Only the first half of the items in *X* and *Y* contained missing values. For each of these variables, missing data could only occur when the corresponding conditioning variable was above the specified cutoff. For the rows that satisfied the cutoff condition, each item was was set to be missing with a certain probability; missingness for all the items was created independently rather than jointly, so that many different missing data patterns would be possible. The population missing rate in each of the items was assignedto one of the two conditions, *p*_*mis*_ = .15 or *p*_*mis*_ = .25, which resulted in an overall missing rate of 7.5% and 12.5%.

In order to examine the effect of variations in the strength of the MAR mechanism, while holding the overall missing rate constant, we defined a variable *p*_cutoff_, which determined the probability that a data point is missing when a value is beyond some specified cutoff. Thus, a higher *p*_cutoff_ leads to a stronger MAR mechanism. For example, when *p*_cutoff = 1_, the MAR mechanism becomes deterministic, such that data are always missing whenever the conditioning variable is beyond the cutoff. With a very low *p*_cutoff_, the cutoff is lower, and the missing mechanism behaves more like MCAR than MAR. When *p*_*mis*_ = *p*_cutoff_, the missing mechanism is MCAR. We set *p*_cutoff = .8_ in strong MAR conditions, and *p*_cuoff_ = .3 in weak MAR conditions. Nonlinear MAR conditions followed a similar definition, except there were two symmetrical cutoffs on the conditioning variable, instead of a single cutoff. Thus, data may be missing when the conditioning variable is above the upper cutoff and when it is below the lower cutoff.

For any fixed overall probability of missingness and the strength of the MAR mechanism, the cutoff of the missing data mechanism must be set to the percentile score *q* = 1 − *p*_*mis*_/*p*_cutoff_. For the nonlinear conditions, the percentiles were *q*_*upper*_ = 1 − .5*p*_*mis*_/*p*_cutoff_ and *q*_*lower*_ = 1 − *q*_*upper*_. These percentile scores were then converted into *z*-scores. Missing data were created with *p*_*mis*_ probability when the value of the conditioning variable was beyond the titz-score cut-off.

### Dependent measures

In order to compare ACML, SL-FIML, MI and TSML, we examined their performance in terms of convergence, bias, relative efficiency, and 95% confidence interval (CI) coverage of the estimated unstandardized regression coefficient, $\hat{\beta}$. Bias is defined as the estimate minus the true value (i.e., raw bias), averaged across all converged replications. Relative efficiency is defined as the ratio of empirical standard error (ESE) of the regression coefficient obtained by each method, compared against TSML. For *n* replications with estimates ${\hat{\beta}}_k$, where *k* = 1, …, *n*, ESE is defined as

$$ ESE=\sqrt{\frac{1}{n-1}\sum \limits_{k=1}^n{\left(\hat{\beta_k}-\overline{\hat{\beta}}\right)}^2} $$

A high ESE indicates the method has a tendency to produce highly varied results from sample to sample. Since the current study aims to examine the performance of the new TSML approach, relative efficiency is defined as an ESE ratio of each method’s ESE over the ESE of TSML. A ratio greater than 1 implies TSML estimates are more efficient (have smaller ESEs). Finally, coverage is the percentage of replications in which the 95% confidence interval (CI) of $\hat{\beta}$ contained the true parameter value.

### Software implementation

All analyses except multiple imputation were conducted using the *lavaan* package (v0.5–23.1097; Rosseel, 2012) in *R* (R Core Team, 2016) and custom code for TSML adapted from Savalei and Rhemtulla (2017).^4^

Multiple imputation was carried out in the *mice* package, with 20 imputations each run, using predictive mean matching as the imputation method in the continuous conditions, and logistic regression in the binary conditions. Mean composites were then created from each imputed dataset. These mean composites were then analyzed using the *runMI* function in the *semTools* package using the same model as ACML. This function produces the pooled regression coefficient estimates and pooled standard errors (Rubin, 1987).

## Results

The performance of each method under every condition can be found in the Supplementary Materials,^5^ which includes tables for raw bias, relative bias, relative efficiency, coverage, and the root mean squared error. Due to space limitations, here we focus on the two conditions where we would expect to see the smallest and the biggest differences between the methods. These are, respectively, Model 1 with 7.5% of overall missingness, where the regression coefficient was medium in size, and the scale item intercorrelations and means were all the same, and Model 8 with 12.5% of overall missingness, where the regression coefficient is high, and each item has different item intercorrelations and item means for the first and second half.

### Convergence of the EM algorithm

The TSML approach can break down during Stage 1 if the saturated model does not converge under the EM algorithm. That is, the FIML estimates of means and covariances sometimes cannot be obtained. In this study, nonconvergence of TSML due to the failure ofsaturated FIML at Stage 1 is very notable at *N* = 50, ranging from about 10% to 50% when the overall missing rate is at 12.5% (see Table 2). While SL-FIML sometimes also encounters nonconvergence, it does so to a much lesser degree, ranging from about 5% to 10% in the same conditions. Because SL-FIML works with composites instead of raw items, there is a significant reduction in the dimensions of the covariance matrix. Convergence of TSML improves significantly when the overall missing rate is 7.5%. At a sample size of 100, TSML encountered virtually no convergence issues in any condition. In the discrete conditions, nonconvergence becomes a severe issue for TSML at *N* = 50 with 12.5% overall missingness, with nonconvergence frequently in the 30%–40% range in both the binary item conditions and the 4-level ordinal conditions (See Table 3). However, nonconvergence drops below 10% if either the missing rate is lowered to 7.5%, or the sample size is increased to *N* = 100. These results suggest that practical applications of TSML may require the use of more sophisticated implementations of the EM algorithm, especially for small sample sizes. In the following sections, SL-FIML and TSML performance is reported based on the average of all converged runs.

**Table 2:** EM Convergence for Continuous Items

Method	Missing	Model	MCAR	S.L. MAR	S.NL. MAR	W.L. MAR	W.NL. MAR
TSML	7.5%	1	54	1	16	80	38
		2	83	0	6	18	29
		3	10	0	3	14	7
		4	13	0	6	6	1
		5	102	0	28	86	37
		6	95	1	4	80	40
		7	32	1	2	10	23
		8	28	1	6	24	14
	12.5%	1	386	268	188	510	318
		2	349	268	180	486	327
		3	135	98	65	187	86
		4	123	115	81	150	95
		5	377	257	194	537	354
		6	401	281	212	537	327
		7	182	179	134	262	146
		8	179	179	109	241	179
SL-FIML	7.5%	1	0	0	0	0	0
		2	0	0	0	0	0
		3	0	0	0	0	0
		4	0	0	0	0	0
		5	0	0	0	0	0
		6	0	0	0	0	0
		7	0	0	0	0	0
		8	0	0	0	0	0
	12.5%	1	86	0	13	42	83
		2	103	0	16	51	86
		3	78	0	1	52	94
		4	78	0	6	34	83
		5	81	0	8	47	72
		6	81	0	7	52	67
		7	87	0	1	39	62
		8	87	0	2	31	91

*Note.* The number of runs where the EM algorithm did not converge (out of 1000 replications) during Stage 1 of TSML and during SL-FIML at *N* = 50. S = Strong, W = Weak, L = Linear, NL = Nonlinear. All other conditions had close to or less than 1

**Table 3:** EM Convergence for Ordinal Items

	Model	MCAR	S.L. MAR	S.NL. MAR	W.L. MAR	W.NL. MAR
TS	1	485	240	556	509	497
2 Levels	2	492	251	509	502	535
	3	528	145	497	484	521
	4	503	150	513	494	527
	5	465	190	476	450	473
	6	438	171	472	468	464
	7	500	85	458	500	490
	8	501	105	468	487	497
4 Levels	1	490	234	404	499	451
	2	468	234	424	472	441
	3	471	181	347	501	468
	4	478	156	404	510	456
	5	422	174	375	453	407
	6	434	136	368	453	421
	7	457	111	373	473	453
	8	443	114	329	496	432
SL-FIML	1	43	0	6	28	35
2 Levels	2	42	0	7	31	37
	3	37	0	2	20	30
	4	47	0	1	24	33
	5	53	0	2	19	46
	6	52	0	3	30	41
	7	38	0	0	28	39
	8	42	0	2	13	51
4 Levels	1	23	0	5	12	29
	2	22	0	8	18	30
	3	23	0	0	18	20
	4	39	0	2	16	32
	5	23	0	4	27	32
	6	40	0	1	10	31
	7	39	0	1	8	33
	8	40	0	1	14	27

*Note.* The number of runs where the EM algorithm did not converge on categorical items (out of 1000 replications) during Stage 1 of TSML and during SL-FIML at *N* = 50 and missing at 12.5%. S = Strong, W = Weak, L = Linear, NL = Nonlinear. TSML and SL-FIML showed comparable or higher nonconvergence at 7.5% in the ordinal conditions compared to the continuous conditions, but the nonconvergence is below 10% in all cases. All other conditions had close to or less than 1% nonconvergence (10 out of 1000 runs)

### Continuous item conditions

#### Results under MCAR

**Fig. 1:**
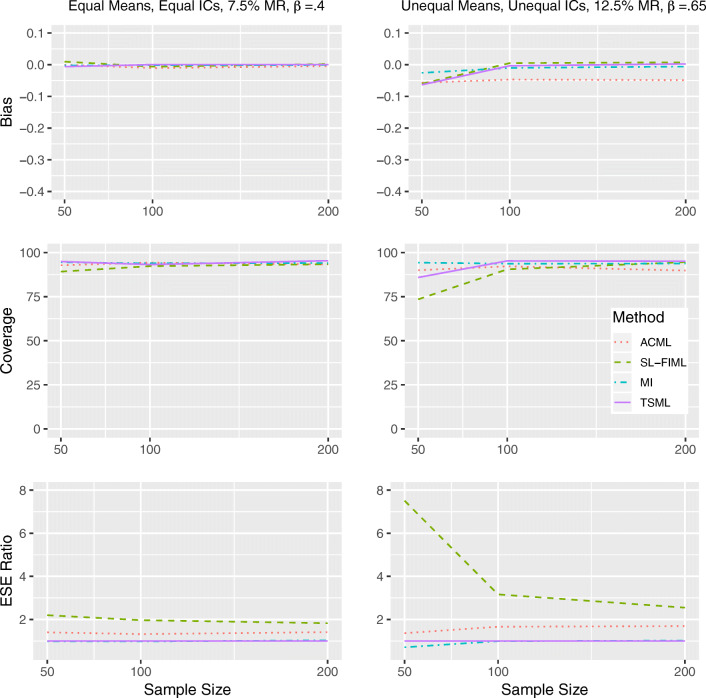
Performance comparison for continuous items, under MCAR in Model 1 (left) and Model 8 (right). IC: Item intercorrelations. MR: Missing rate. *β*: The unstandardized regression coefficient. Empirical standard error (ESE) ratio: The relative efficiency of each method is measured against TSML, by taking the ESE of that method, divided by the ESE of TSML.

The top, middle, and bottom panels of Fig. 1 show the bias, coverage, and relative efficiency of the unstandardized regression coefficient estimates. As a reminder, Model 1 in the study contains equal item intercorrelations, equal item means, and a medium regression coefficient (more specifically, *ρ*_1_ = *ρ*_2_ = .49, *μ*_1_ = *μ*_2_ = 0, *β* = .40). Model 1 represents the intersection of the most ideal conditions suggested by previous studies. In the left panels, we show how each method performs under Model 1 with 7.5% missing. Although it is unrealistic, it acts as a useful benchmark: Any performance issue under such conditions should be deemed unacceptable. In contrast, Model 8 contains unequal item intercorrelations, unequal item means, and a high regression coefficient (*ρ*_1_ = .25, *ρ*_2_ = .64, *μ*_1_ = 0, *μ*_2_ = .5, *β* = .65). This is a more complex model that represents a more realistic situation, with a higher potential for bias. The right panels present Model 8 with 12.5% missing, in order to demonstrate how methods may be expected to break down in practice.

We can see that all methods show comparable performances overall for Model 1 with a 7.5% missing rate. The only notable difference is that SL-FIML, which is the least efficient approach, has a less than ideal coverage rate of 89.2% at *N* = 50. InModel 8 with a 12.5% missing rate, all methods show negative bias at *N* = 50, with MI showing the least bias. For SL-FIML and TSML, the notable bias appears to be the result of convergence issues, as the bias disappears entirely once the sample size reaches *N* = 100. In ACML, however, the bias persists at all levels of sample size conditions, leading to poor coverage. While SL-FIML and TSML both show good coverage at *N* = 200, SL-FIML is once again less efficient. Although it produces unbiased estimates, SL-FIML shows comparable coverage to ACML at *N* = 100. MI and TSML show comparable performances at *N* = 100 and *N* = 200.

#### Results under strong MAR

**Fig. 2:**
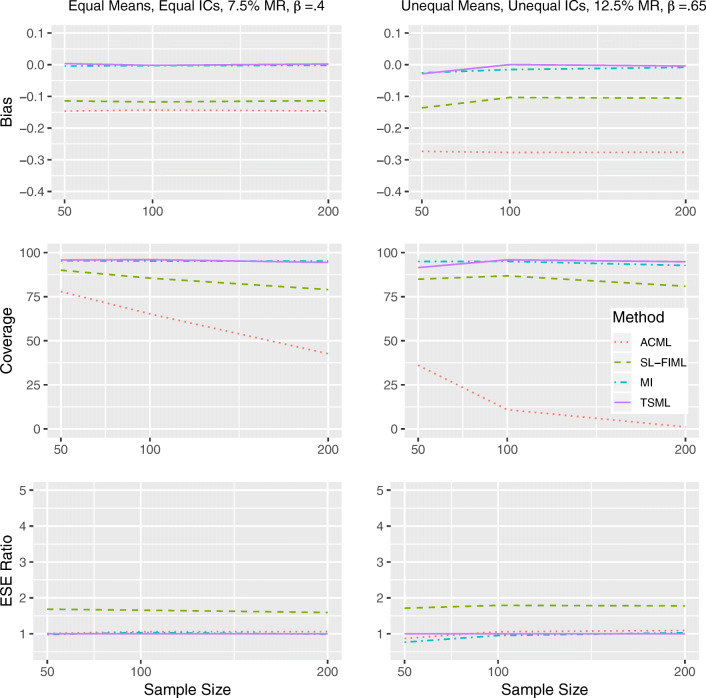
Performance comparison for continuous items, under strong linear MAR in Model 1 (left) and Model 8 (right). IC: Item intercorrelations. MR: Missing rate. *β*: The unstandardized regression coefficient. Empirical standard error (ESE) ratio: The relative efficiency of each method is measured against TSML, by taking the ESE of that method, divided by the ESE of TSML.

**Fig. 3:**
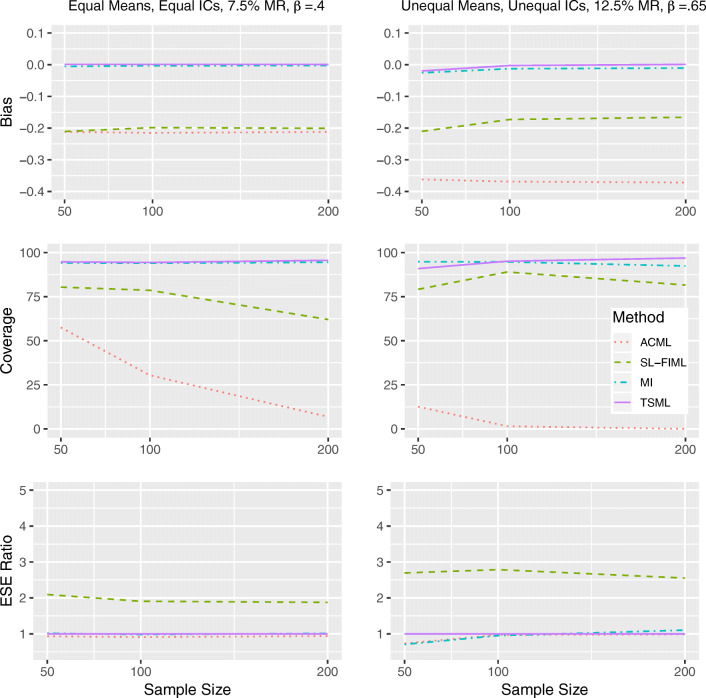
Performance comparison for continuous items, under strong nonlinear MAR in Model 1 (left) and Model 8 (right). IC: Item intercorrelations. MR: Missing rate. *β*: The unstandardized regression coefficient. Empirical standard error (ESE) ratio: The relative efficiency of each method is measured against TSML, by taking the ESE of that method, divided by the ESE of TSML.

In Figs. 2 and 3, we see that TSML and MI are largely unbiased under strong MAR conditions. For Model 8 with 12.5% missing at *N* = 50, TSML encounters convergence issues, which results in slightly lower levels of coverage (91.5% and 90.9%). However, the method achieves ideal coverage as long as the sample size is larger. While SL-FIML shows the exact same pattern of underestimation, it also produces higher standard errors in those cases, which leads to slightly better coverage. ACML and SL-FIML produce notable underestimates for both models, with a larger bias in nonlinear than linear conditions. Although the bias is consistent across all sample sizes, the coverage becomes worse as these methods become more confidentin the incorrect estimates. At *N* = 200, the ACML coverage for Model 8 is practically 0. The SL-FIML performance, while not as poor, is also unsatisfactory.

#### Results under weak MAR

**Fig. 4:**
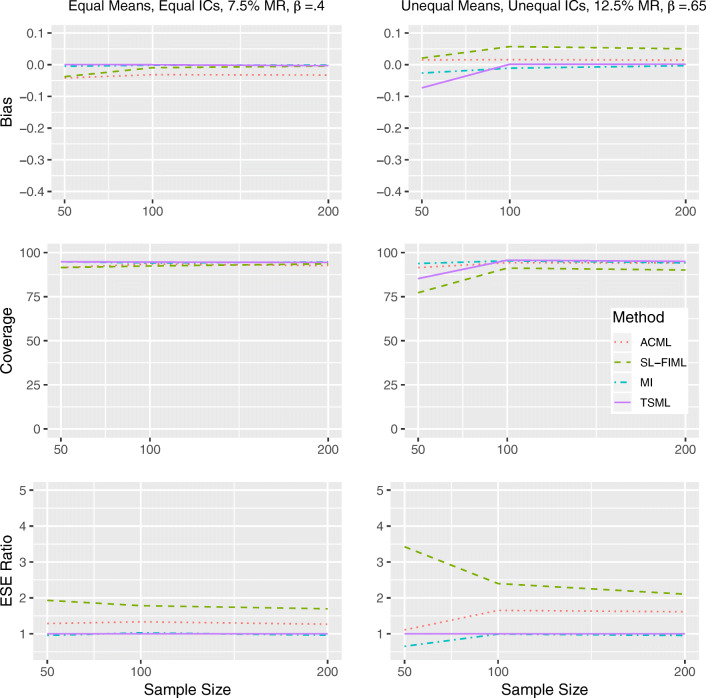
Performance comparison for continuous items, under weak linear MAR in Model 1 (left) and Model 8 (right). IC: Item intercorrelations. MR: Missing rate. *β*: The unstandardized regression coefficient. Empirical standard error (ESE) ratio: The relative efficiency of each method is measured against TSML, by taking the ESE of that method, divided by the ESE of TSML.

**Fig. 5:**
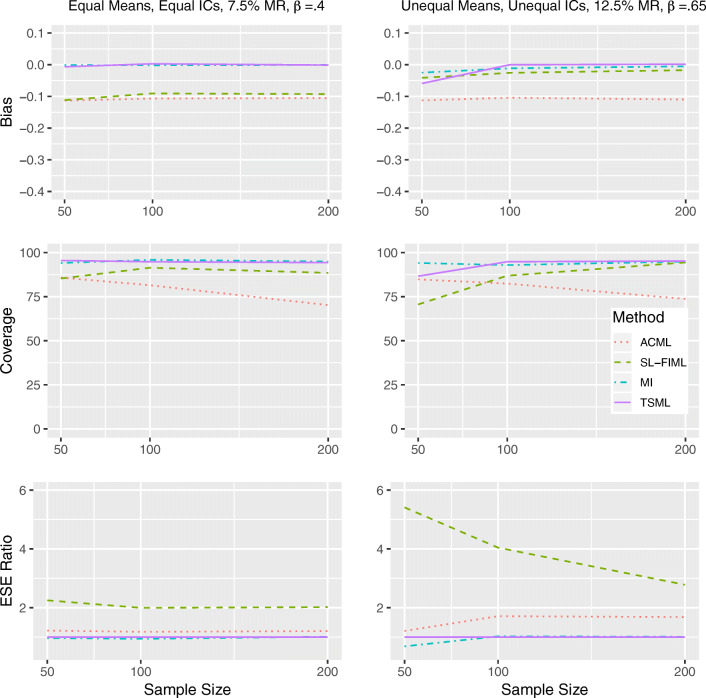
Performance comparison for continuous items, under weak nonlinear MAR in Model 1 (left) and Model 8 (right). IC: Item intercorrelations. MR: Missing rate. *β*: The unstandardized regression coefficient. Empirical standard error (ESE) ratio: The relative efficiency of each method is measured against TSML, by taking the ESE of that method, divided by the ESE of TSML.

Similar to the strong MAR conditions, MI and TSML show similar performance under both linear and nonlinear weak MAR (Fig. 4 and Fig. 5), with the exception of Model 8 at 12.5% missing, where TSML runs into convergence issues at *N* = 50. For weak MAR conditions with convergence issues, the TSML coverage are 85.2% (linear) and 86.6% (nonlinear).

Under linear weak MAR, SL-FIML and ACML are both inefficient, even for Model 1 at 7.5% missing. While SL-FIML is only slightly biased at *N* = 50, ACML consistently underestimates the regression coefficient. For Model 8 with 12.5% missing, SL-FIML overestimates the regression coefficient, showing both bad coverage and low efficiency. ACML also overestimates the parameter in the same model, but to a lesser extent. The ACML coverage is similar to that of MI and TSML due to its high standard error, but the approach suffers in efficiency as a result. Under nonlinear weak MAR, ACML shows similar performance issues as strong nonlinear MAR in both models. The method produces a consistent underestimate, which leads to worsening coverage as the sample size grows. SL-FIML produces underestimates under Model 1, with mediocre coverage. Under Model 8, it produces more accurate estimates, although its coverage is low even at *N* = 100, due to its inefficiency.

### Discrete item conditions

**Fig. 6:**
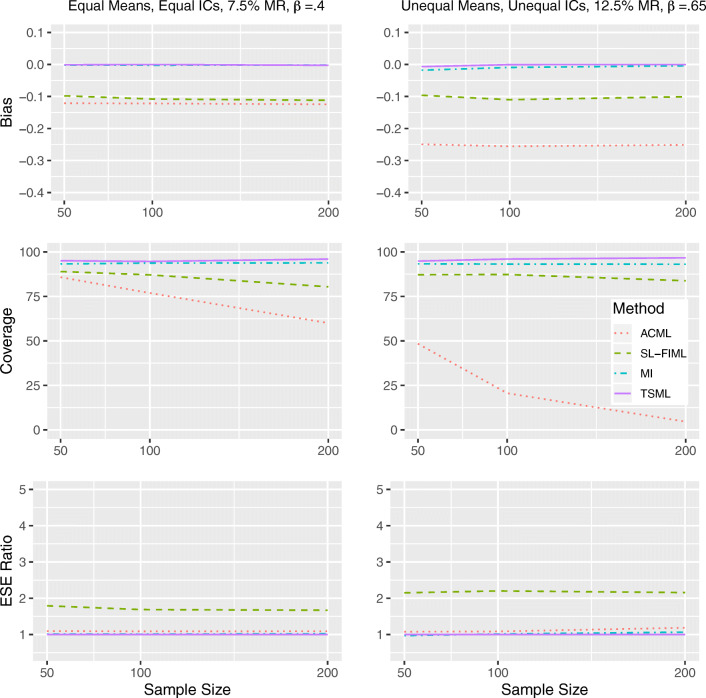
Performance comparison for binary items, under strong linear MAR in Model 1 (left) and Model 8 (right). IC: Item intercorrelations. MR: Missing rate. *β*: The unstandardized regression coefficient. Empirical standard error (ESE) ratio: The relative efficiency of each method is measured against TSML, by taking the ESE of that method, divided by the ESE of TSML.

**Fig. 7:**
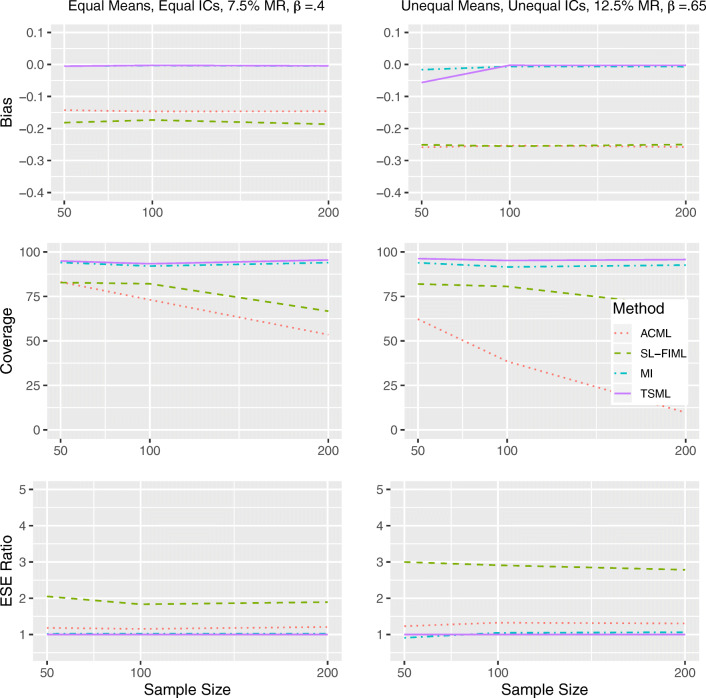
Performance comparison for binary items, under strong nonlinear MAR in Model 1 (left) and Model 8 (right). IC: Item intercorrelations. MR: Missing rate. *β*: The unstandardized regression coefficient. Empirical standard error (ESE) ratio: The relative efficiency of each method is measured against TSML, by taking the ESE of that method, divided by the ESE of TSML.

The performance of each method with ordinal items is largely similar to the performance with continuous items, except at *N* = 50 and overall missing rate of 12.5%. In these conditions, TSML encounters a large number of convergence issues, resulting in biased estimates and unacceptably wide confidence intervals. The bias is less in the strong linear MAR condition than other conditions, but the relative bias can reach minus 20% under weak linear MAR. The performance of ACML improves slightly under the MAR conditions. For direct comparison to the continuous conditions, Figs. 6 and 7 show the performance of ACML, SL-FIML, MI, and TSML under the strong linear MAR and strong nonlinear MAR with binary items. It is remarkable that TSML does well with discrete data (with the exception of the conditions outlined above) because this method does assume continuous normal data at the item level. MI shows a slightly lower coverage overall, occasionally drops down to 92%.

## Discussion

When data are missing at the item level but the model is at the composite level, the usual FIML approach to the treatment of missing data is not easily available. The current study compared an alternative analytical approach known as TSML (Yuan & Bentler, 2000), which has recently been extended to item-level missing data (Savalei & Rhemtulla, 2017), and two procedures popular among applied researchers, ACML and SL-FIML, in the context of univariate regression. Item-level MI was also included as a comparison method. A wide array of simulation conditions was studied, including different missing data rates, types of missing data mechanism, population models, sample size, and type of items (continuous, binary, or 4-category). In accordance with theoretical expectations, TSML and MI performed best, and ACML and SL-FIML exhibited serious problems under some conditions.

We recommend the TSML or the MI approach for item-level missingness. The MI approach produced unbiased and efficient estimates and good coverage across all study conditions. TSML approach had essentially identical performance except in conditions where the EM algorithm was not able to converge during Stage 1 (i.e., when estimating the saturated covariance matrix of the items) in many replications. These convergence issues occurred at the smallest studied sample and with the highest rate of missingness. Future research will investigate whether the convergence of the EM algorithm can be improved by adjusting the starting values or using different software packages to see if a more effective implementation exists.

While it was theoretically expected that MI and TSML would perform similarly with continuous data, as they are expected to be asymptotically equivalent when the number of imputations is large, it was remarkable that TSML did very well with binary and 4-category data as well, performing indistinguishably from categorical MI (outside of problems with convergence in select conditions). Existing recommendations for use of continuous methods with categorical data is that the items should have at least 5–7 categories (Rhemtulla et al., 2012). It appears, however, that when the model is at the composite level, items comprising the composites can be treated as continuous even when they have fewer categories. Similar results have been found in the context of MI, which are consistent with previous research (Wu, Jia, & Enders, 2015).

The key difference between the two recommended methods is that TSML, as an analytical alternative, provides the same answer every time, while MI, as a simulation-based approach, produces different answers. Furthermore, MI can take up a substantially longer computational time, especially with ordinal data, and it is not always straightforward to implement for applied researchers. Thus, we believe researchers may prefer the analytical alternative (TSML) to conducting MI when this method converges. For researchers interested in applying the TSML approach, an implementation in *R* is available on OSF.^6^ This online material also includes a *Shiny* app (RStudio, Inc, 2013).

When researchers encounter item-level missing data, they may be tempted to use the ACML approach (i.e., person-level imputation) or the SL-FIML approach due to their convenience. However, the current study adds to the body of evidence that suggests that the ACML approach comes with substantial risks (e.g., Schafer & Graham, 2002; Enders, 2003; Mazza et al., 2015). When data are MCAR, the bias in the regression coefficient by ACML is reasonably small, and may not have a serious impact if the regression coefficient is low and the missing rate is also very low. However, even small underestimation can lead to unacceptable coverage with a larger missing rate or high regression coefficient, especially when the item intercorrelations are not equal. More critically, under strong MAR, ACML may drastically underestimate the regression coefficient, and have very poor coverage as a result. Under weak MAR, ACML has reasonable performance in the linear-MAR condition, but quite poor performance in the nonlinear-MAR condition. While SL-FIML is able to produce unbiased estimates under MCAR, it is inefficient. It also has low coverage at smaller sample sizes, which suggests that while the long run average estimate is unbiased, the loss of information due to discarding a lot of data is too great to produce accurate standard errors at small sample sizes. Under MAR conditions, SL-FIML also tends to underestimate the regression coefficient and show inadequate coverage. Another potential rationale for the application of ACML of SL-FIML is that item scores are typically ordinal in nature. Intuitions may suggest that ACML and SL-FIML would alleviate this problem by creating a total score that more closely approximates a continuous score. However, the results of the current study do not support this intuition. Although ACML and SL-FIML performed slightly better with binary and 4-category data, their performances were still unsatisfactory, suffering from the same patterns of biased estimates, low coverage, and low efficiency.

The current study only examined univariate regression; however, because the theoretical properties of TSML (consistency under MAR, high efficiency) are better than those of ACML and SL-FIML, we expect that TSML will outperform these other methods in the context of multiple regression or other models. Of course, the degree to which bias and loss of efficiency will manifest in more complex models can differ. In particular, univariate regression without auxiliary variables is the worst-case scenario for SL-FIML, as the conditioning variable is always deleted at the item-level when data are missing, resulting in MNAR missingness. If all conditioning variables happen to be outside of composite scores with missing data, SL-FIML should provide unbiased estimates, with only a loss of efficiency. More complex models will provide the opportunity to investigate the performance of the method between the worst-case scenario and the ideal scenario.

All methods discussed in this paper assume a linear relationship between the predictor and the outcome variable. In the presence of nonlinear effects, each approach must modify the model accordingly. In order to include a nonlinear effect (such as a quadratic effect) under the TSML or MI approaches, the nonlinear terms (e.g., squares of each item and cross-products of items on the same composite) must be added to the model during Stage 1 (for TS) or during the imputation stage (for item-level MI). With MI, while it may seem that nonlinear effects can be added during the analysis stage, such an approach is not recommended (Seaman, Bartlett, & White, 2012).

In the current study, the composites were not explicitly conceptualized as reflective, though the item correlation structures were consistent with reflective models in some models (equal inter-correlations). TSML can be applied to composites that are either formative or reflective. This differs from the previous study which applied the method in the context of SEMs with parcels (Savalei & Rhemtulla, 2017). As regression models are very common in psychological research, we believe our application has high relevance for applied researchers.

## Open practices statement

The code used to perform data generation and analyses in this simulation study is available on OSF: https://osf.io/8u9fm/

## Footnotes

1 Here we assume no participant is missing all of the items on any of the composites, so that there is no missing data at the composite level. However, in practice this is not a problem for the method, because FIML rather than complete data ML can also be run on the composite scores should some of them be missing.

2 available at https://osf.io/8u9fm/

3 In our models, the standardized and unstandardized regression coefficients were numerically identical in the population, because the predictor and the outcome variable had the same population variance. The results reported below are based on the sample unstandardized regression coefficients.

4 Available at https://osf.io/8u9fm/

5 Available at https://osf.io/8u9fm/

6 https://osf.io/8u9fm/
